# Darbepoetin alfa for the treatment of anemia in myelodysplastic syndromes: a post-marketing surveillance study in Japan

**DOI:** 10.1007/s12185-026-04227-w

**Published:** 2026-05-18

**Authors:** Yasuyoshi Morita, Yukie Tsuji, Tomoharu Yasukawa, Kinuko Mitani

**Affiliations:** 1 Department of Hematology and Rheumatology, Kindai University Faculty of Medicine, 377-2 Ohno-Higashi, Osaka-Sayama, 589-8511 Osaka Japan; 2 Pharmacovigilance Department, Kyowa Kirin Co., Ltd., Tokyo, Japan; 3 Department of Medicine, Dokkyo Medical University, Tochigi, Japan

**Keywords:** Acute myeloid leukemia, Anemia, Darbepoetin alfa, Myelodysplastic syndromes, Post-marketing surveillance

## Abstract

Post-marketing surveillance was conducted in Japan (2015–2024, UMIN000056049) to evaluate the long-term safety and effectiveness of darbepoetin alfa for anemia with myelodysplastic syndromes (MDS) for 5 years after treatment initiation. Among 1834 patients (median age: 79.0 years), the distribution of International Prognostic Scoring System (IPSS) risk categories was: Low, 39.3%; Intermediate-1, 45.1%; Intermediate-2, 7.7%; and High, 2.8%. The median (range) administration duration, dosing interval, and weekly dose were 330.0 (1–1863) days, 1.7 (0.2–41.5) weeks, and 240.0 (30.0–250.0) µg, respectively. Treatment discontinuation reasons included inadequate response (36.9%) and adverse events (AEs) (31.2%). AEs were reported in 75.1% and included pneumonia (16.6%) and transformation to acute myeloid leukemia (9.0%). Mean hemoglobin concentration (n = 1821) increased from 7.6 g/dL at baseline to > 9.0 g/dL after 52 weeks. Red blood cell transfusion volume decreased by ≥ 50% compared with baseline in 65.2% of patients. In transfusion-dependent patients (n = 909), the highest transfusion-independence rate was 40.9% at year 4–5. Five-year overall/leukemia-free survival rates (n = 1826/1814) by IPSS risk group were: Low, 49.5%/48.8%, Intermediate-1, 31.1%/30.2%, Intermediate-2, 11.9%/10.4%, and High, 0%/0%. Poor prognostic factors for MDS were IPSS risk category, age, chromosomal abnormalities, and platelet count. No new safety or effectiveness concerns were identified.

*Trial registration:* University Hospital Medical Information Network; study ID: UMIN000056049.

**Supplementary Information:**

The online version contains supplementary material available at https://doi.org/10.1007/s12185-026-04227-w.

## Introduction

Myelodysplastic syndromes (MDS) are hematopoietic stem cell neoplasms that initially present with abnormal blood cell morphology (dysplasia) and cytopenia, transforming to leukemia in the late stage [1]. The primary treatment in patients with lower-risk MDS with cytopenic symptoms focuses on the cytopenia, including red blood cell transfusion for anemia, platelet transfusion for thrombocytopenia, and infection control, with the aim of improving these conditions [2]. A more intensive treatment strategy is used to alter the natural course of the disease in high-risk patients because of the higher risk of transformation to leukemia [2].

Cytokine therapy agents such as erythropoietin (EPO) erythropoiesis-stimulating agents (ESAs), and granulocyte colony-stimulating factor are recommended as effective therapeutic options in lower-risk patients with cytopenia [1–4]. ESAs are established therapies with a high level of evidence for anemia associated with lower-risk MDS, according to international textbooks [5] and treatment guidelines in Japan (evidence Ib/IIb [1], category 2A [2]) and overseas (recommended regimen [3], evidence I, A [4]). Darbepoetin alfa (DA) is a long-acting ESA that was approved in Japan in 2014 for the treatment of anemia associated with MDS, at a dosage of 240 µg administered subcutaneously once a week (QW), though the dosage and administration interval for this indication in other countries is 500 µg every 3 weeks [6].

A clinical trial conducted to obtain approval in Japan confirmed the effectiveness and safety of subcutaneous administration of DA at 60, 120, or 240 µg QW in patients with red blood cell transfusion-dependent MDS classified as International Prognostic Scoring System (IPSS) Low or Intermediate-1 risk (n = 52) [7]. The primary endpoint was a major or minor erythroid response at 16 weeks, followed by a 48-week observation period. Patients in the 240-µg group had the highest major response rate (33.3%). DA was well tolerated across all dose groups, with no dose-dependent adverse events (AEs), and 240 µg QW was accordingly considered to be the appropriate starting dose for DA [7]. However, given that only 15 of the 52 patients received the recommended 240 µg QW dose for MDS during the 48-week observation period [7], the clinical data on anemia associated with MDS were limited at the time of approval in Japan. We therefore conducted a post-marketing surveillance (PMS) study of DA in Japan to collect additional real-world data. This PMS study aimed to evaluate the long-term safety and effectiveness of DA for anemia associated with MDS over a 5-year period under real-world clinical conditions.

## Materials and methods

### Study design, patients, and treatment

This observational PMS (UMIN000056049) was conducted in Japan from February 2015 to October 2024 in compliance with the Japanese Good Post-marketing Study Practice (GPSP). Approval for this PMS was obtained from the Research Ethics Review Committee of Kyowa Kirin Co., Ltd. This was an observational study designed to evaluate an approved drug and indication, and the need for informed consent was waived by the GPSP because the data collection was completely anonymized. Eligible patients were those with anemia associated with MDS who received DA in routine clinical practice. The observation period was 5 years after DA initiation.

Consent for this publication was obtained from each participating site, and only the permitted data for publication were included in this manuscript.

### Outcomes

#### Safety

Any AE that could not be definitively excluded as causally related to DA was classified as an adverse drug reaction (ADR). AEs and ADRs were coded according to the Medical Dictionary for Regulatory Activities, Japanese Version 27.0 (MedDRA/J). Seriousness was classified according to the International Conference on Harmonisation of Technical Requirements for Registration of Pharmaceuticals for Human Use (E2D) guidelines.

#### Effectiveness

Changes in mean hemoglobin (Hb) concentration up to 260 weeks after DA initiation were summarized. The number and percentage of patients requiring blood transfusions each year after DA initiation were summarized according to whether or not they had received transfusions during the 56 days prior to the evaluation. The erythroid response was evaluated based on changes in red blood cell transfusion volume during the 56 days or Hb concentration following DA initiation, and classified as: minor response, red blood cell transfusion volume during the last 56 days decreased by ≥ 50% from baseline; major response, patients no longer require red blood cell transfusion (i.e., achieve transfusion independence) for at least the last 56 consecutive days and highest Hb concentration during the transfusion-free period increased by ≥ 1.0 g/dL from baseline; and minor response or greater, patients with either minor or major response. The cumulative erythroid response rates up to 260 weeks after DA initiation were summarized separately for a minor response or greater and for a major response. The clinical global impression was also evaluated annually by attending investigators as “effective,” “ineffective,” or “unable to determine” for every year for up to 5 years after DA initiation.

#### Overall survival (OS) and leukemia-free survival (LFS)

Since clinical trials conducted to obtain approval in Japan had not evaluated long-term survival outcomes or acute myeloid leukemia (AML) progression following adequate duration of DA administration, this PMS established OS and LFS as priority survey items. OS and LFS data were collected for every year for up to 5 years after DA initiation for all patients (including patients who discontinued DA treatment), excluding those lost to follow-up.

### Statistical analysis

The number (%) of patients showing an erythroid response (minor, major) was calculated with 95% confidence intervals (CIs) according to IPSS risk categories. Erythroid responses by time point (at weeks 8 and 52) and by subgroups (prior ESA treatment, pre-treatment EPO concentration, and major karyotype) were also calculated. The cumulative erythroid response curves were plotted, and the median time to achieving each response, along with 95% CI, was estimated for a minor response or greater and for a major response, respectively. OS and LFS curves were plotted using the Kaplan–Meier method. Events were “death” or “death or transformation to AML.” The number of patients analyzed and number of events were calculated at each time point, and the median survival time with 95% CI was estimated for each subgroup. Results were compared between subgroups using log-rank tests. The number of patients at risk and rates of OS and LFS with 95% CI were also shown by subgroups and by number of years after DA initiation (1–5 years). The subgroups were defined by IPSS risk category (Low, Intermediate-1, Intermediate-2, High), chromosomal abnormalities (no, yes), highest Hb concentration after DA treatment (≥ 10 g/dL, 9 to < 10 g/dL, 8 to < 9 g/dL, < 8 g/dL), and erythroid response (major, minor, no). Prognostic factors for death and transformation to AML were analyzed using a multivariable Cox proportional hazards model targeting cases with no missing values in the explanatory variables, with patient background factors, including variables with p < 0.05 in univariable analyses. The patient background factors included IPSS risk classification, age, and chromosomal abnormalities, baseline Hb concentration, neutrophil count, and platelet count, and the presence or absence of a history or comorbidity of malignant tumors. Hazard ratios and their 95% CI were obtained from the model, with the CIs based on the standard errors of the coefficients. Statistical significance was assessed using the Wald χ^2^ test. No imputation was made for missing data. Statistical analysis was carried out using SAS version 9.4 (SAS Institute Inc., Cary, NC, USA).

## Results

### Patient disposition and characteristics

Patient disposition is shown in Supplementary Fig. 1. A total of 2,091 patients were registered from 460 sites between February 2015 and June 2019. Of these, 126 patients for whom survey forms were not available, 123 patients without permission to publish the data, and three patients who did not receive DA were excluded, and 1,839 patients were finally included in the analysis population. The safety analysis population included 1,834 patients, after excluding a further five patients with unevaluable data. The effectiveness analysis population comprised 1,821 patients, after excluding an additional 13 patients without MDS from the safety analysis population. Prognostic factors for mortality were analyzed in 1,826 patients, after 13 patients without MDS were excluded, and factors for death and transformation to AML were analyzed in 1,814 patients, after excluding 25 patients with unevaluable data for the analysis (n = 15) or an incorrect diagnosis of MDS (n = 13) from the analysis population.

The baseline characteristics of the safety analysis population are shown in Table 1. Patients in the IPSS Intermediate-2 (7.7%) and High (2.8%) risk categories, for whom effectiveness and safety had not been confirmed at approval, were also included in this PMS.

**Table 1:** Patient characteristics

Characteristic	Classification	Safety analysis population (n = 1834)	
**Sex**	Male	1136	(61.9%)
	Female	698	(38.1%)
**Age (years)**	< 75	580	(31.6%)
	≥ 75	1254	(68.4%)
(n = 1834)	Median (range)	79.0 (22–101)	
**PS (ECOG)**	0	546	(29.8%)
	1	839	(45.7%)
	2	231	(12.6%)
	3	112	(6.1%)
	4	22	(1.2%)
	Unknown/not recorded	84	(4.6%)
**Reason for use (counted duplicates)**	Anemia associated with MDS	1822	(99.3%)
	Others	20	(1.1%)
**IPSS risk categories immediately before DA initiation**	Low	720	(39.3%)
	Intermediate-1	827	(45.1%)
	Intermediate-2	141	(7.7%)
	High	52	(2.8%)
	Unknown/not recorded	94	(5.1%)
**IPSS-R risk categories immediately before DA initiation**	Very low	183	(10.0%)
	Low	573	(31.2%)
	Intermediate	448	(24.4%)
	High	164	(8.9%)
	Very high	110	(6.0%)
	Unknown/not recorded	356	(19.4%)
**WHO categories (ver.3) immediately before DA initiation**	RA	472	(25.7%)
	RCMD	649	(35.4%)
	RARS	106	(5.8%)
	RCMD-RS	33	(1.8%)
	RAEB-1	158	(8.6%)
	RAEB-2	75	(4.1%)
	MDS-U	56	(3.1%)
	5q-syndrome	21	(1.1%)
	Unknown/not recorded	264	(14.4%)
**Treatment history of MDS**	No	656	(35.8%)
	Yes	1172	(63.9%)
	Unknown/not recorded	6	(0.3%)
**Treatment history of MDS (types) (counted duplicates)**	Red blood cell transfusion	930	(50.7%)
	Platelet transfusion	149	(8.1%)
	ESA preparations	123	(6.7%)
	G-CSF preparations	59	(3.2%)
	Iron chelator	132	(7.2%)
	Vitamin therapy	178	(9.7%)
	Immunosuppressive therapy	122	(6.7%)
	Chemotherapy	27	(1.5%)
	Hematopoietic stem cell transplantation	4	(0.2%)
	Lenalidomide	20	(1.1%)
	Azacitidine	168	(9.2%)
	Others	169	(9.2%)
Treatment history with ESA	No	1705	(93.0%)
	Yes	123	(6.7%)
	Unknown/not recorded	6	(0.3%)
Chromosomal abnormalities	No	1166	(63.6%)
	Yes	662	(36.1%)
	Unknown/not recorded	6	(0.3%)
Chromosomal abnormalities (karyotypes) (counted duplicates)	+ 8	110	(6.0%)
	-7 or del (7q)	78	(4.3%)
	-5 or del (5q)	91	(5.0%)
	-5 or del (5q) alone	24	(1.3%)
	-5 or del(5q) with one other than -7 or del(7q)	15	(0.8%)
	-5 or del(5q) with one of -7 or del(7q)	3	(0.2%)
	Complex karyotype with -5 or del(5q)	49	(2.7%)
	del(20q)	135	(7.4%)
	-Y	142	(7.7%)
	i (17q) or t (17p)	10	(0.5%)
	-13 or del (13q)	25	(1.4%)
	del(11q)	24	(1.3%)
	del (12p) or t (12p)	8	(0.4%)
	del(9q)	13	(0.7%)
	idic(X)(q13)	2	(0.1%)
	t(11;16)(q23;p13.3)	0	(0.0%)
	t(3;21)(q26.2;q22.1)	0	(0.0%)
	t(1;3)(p36.3;q21.2)	0	(0.0%)
	t(2;11)(p21;q23)	1	(0.1%)
	inv(3)(q21q26.2)	1	(0.1%)
	t(6;9)(p23;p34)	0	(0.0%)
	Complex karyotype (≥ 3 abnormalities)	144	(7.9%)
	Others	341	(18.6%)
Pre-treatment Hb concentration (g/dL)	< 8.0	1049	(57.2%)
	≥ 8.0	588	(32.1%)
	Unknown/not recorded	197	(10.7%)
(n = 1637)	Mean ± SD	7.6 ± 1.3	
Pre-treatment neutrophil count (× 10^3^/µL) (n = 1543)	Mean ± SD	5.1 ± 79.5	
Pre-treatment platelet count (× 10^3^/µL) (n = 1623)	Mean ± SD	155.6 ± 156.8	
Pre-treatment EPO concentration (mlU/mL)	< 500	449	(24.5%)
	≥ 500	104	(5.7%)
	Unknown/not recorded	1281	(69.8%)
(n = 553)	Mean ± SD	499.1 ± 1261.8	
History of red blood cell transfusion within 56 days before DA initiation	No	916	(49.9%)
	Yes	917	(50.0%)
	Unknown/not recorded	1	(0.1%)
Transfusion volume of red blood cell (mL) (n = 913)	Mean ± SD	1073.9 ± 769.4	

*DA* darbepoetin alfa, *EPO* erythropoietin, *ESA* erythropoiesis-stimulating agent, *G-CSF* granulocyte colony stimulating factor, *Hb* hemoglobin, *IPSS* International Prognostic Scoring System, *MDS* myelodysplastic syndromes, *MDS-U* myelodysplastic syndromes unclassifiable, *PS (ECOG)* performance status (Eastern Cooperative Oncology Group), *RA* refractory anemia, *RAEB* refractory anemia with excess blasts, *RARS* refractory anemia with ringed sideroblasts, *RCMD* refractory cytopenia with multilineage dysplasia, *RCMD-RS* RCMD with ringed sideroblasts, *SD* standard deviation

### Status of DA treatment and discontinuation

**Fig. 1:**
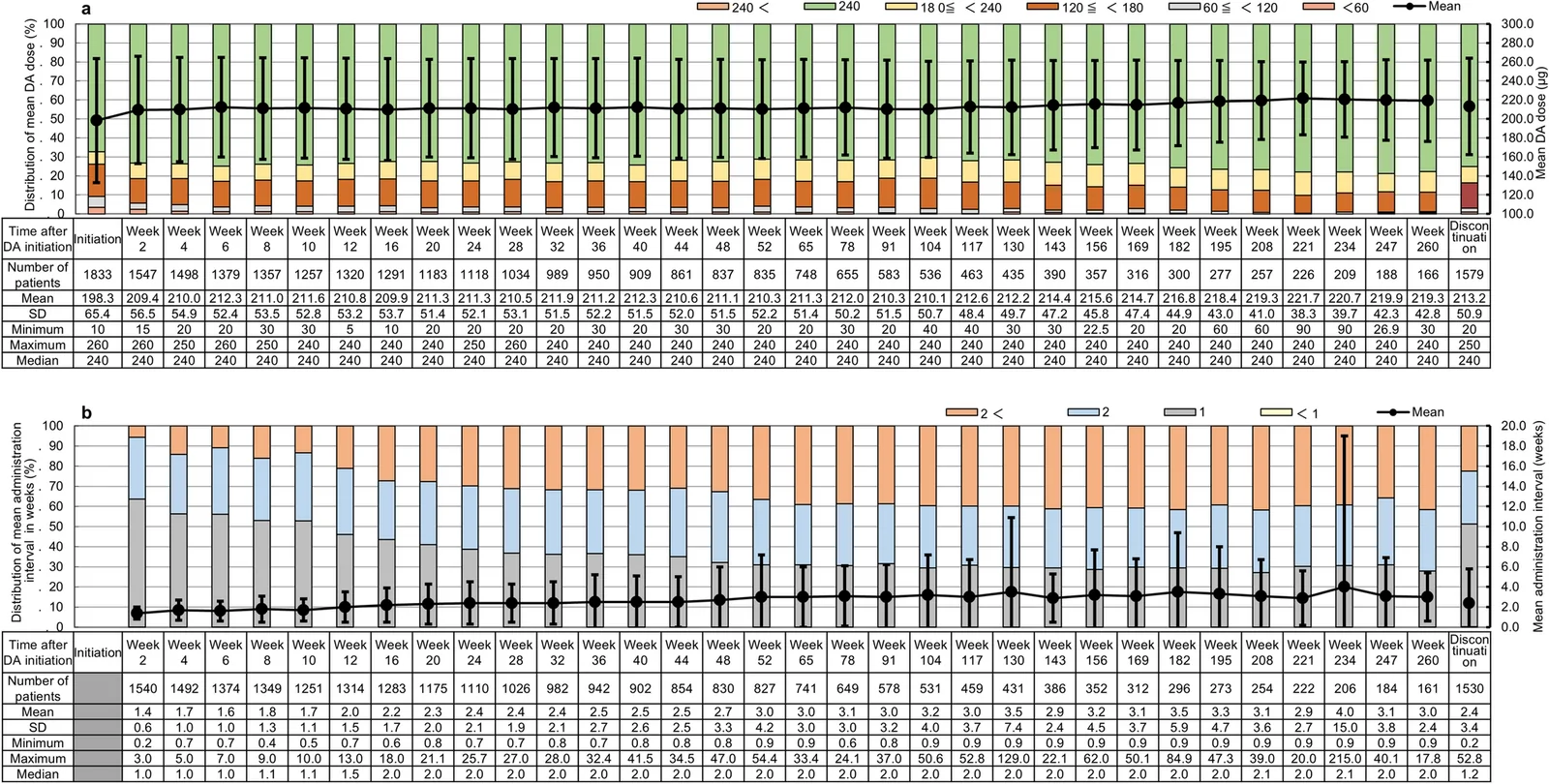
Time course of mean DA dose and its distribution (**a**), and mean administration interval and its distribution (**b**) in patients. The administration status at each time point up to 260 weeks after DA initiation is shown for the safety analysis population. The dose and administration interval are presented as means ± SDs. The administration interval of “1 week” was defined as 7 ± 3 days from the previous DA administration. The distribution of the mean dose (< 60 µg, 60–120 µg, 120–180 µg, 180–240 µg, 240 µg, > 240 µg) and the mean administration interval (< 1 week, 1 week, 2 weeks, and > 2 weeks) are shown by color. Although difficult to discern in the figure, the maximum distribution for the mean dose category “ > 240 µg” (**a**) and for the mean administration interval category “ < 1 week” (**b**) during the observation period were both only 0.2%. *DA* darbepoetin alfa, *SD* standard deviation

The mean dose of DA per administration ranged from 209.4–221.7 µg from weeks 2–260 after DA initiation, with no marked change throughout the observation period (Fig. 1a). The median (range) administration number and dose per week were 0.6 (0.0–1.6) and 240.0 (30.0–250.0) µg, respectively. The mean administration intervals were 1.4 weeks at week 2, but 2.0–2.7 weeks and 3.0–4.0 weeks at weeks 12–48 and 52–260, respectively, indicating a trend toward longer administration intervals in patients receiving long-term treatment (Fig. 1b). The longest mean administration interval (4.0 weeks) was observed at week 234 (Fig. 1b). The median (range) administration period and dosing interval were 330.0 (1–1863) days and 1.7 (0.2–41.5) weeks throughout the observation period, respectively.

DA treatment was discontinued in 86.5% of patients within 5 years, mainly because of inadequate response (36.9%) or AEs (31.2%). Higher IPSS risk was associated with a shorter period until discontinuation. The median (range) times to discontinuation were 267.0 (1–1,825), 360.0 (1–1,825), 269.0 (1–1,800), 141.0 (1–1,289), and 85.5 (6–974) days in All, Low-, Intermediate-1-, Intermediate-2-, and High-risk patients, respectively. Some patients with high IPSS risk continued long-term administration, with maximum times to discontinuation of 1,289 and 974 days in Intermediate-2- and High-risk patients, respectively.

### Concomitant use of MDS treatment

MDS treatment drugs were not used concomitantly with DA in 55.2% (1,013/1,834) of patients, but 44.4% (815/1,834) of patients did receive concomitant MDS drugs and the status was unknown or not recorded in the remaining 0.3% (6/1,834) of patients. The most frequently used concomitant MDS-directed agents (≥ 10%) among the 815 patients were as follows: metenolone acetate, 37.9% (309/815); azacitidine, 22.3% (182/815); menatetrenone, 15.1% (123/815); cyclosporine, 12.9% (105/815); and prednisolone, 12.4% (101/815). Concomitant granulocyte colony-stimulating factor was used in 5.3% (43/815) of patients, of whom 32.6% (14/43) started concomitant use within 16 weeks after DA initiation. Concomitant red blood cell transfusion was performed in 79.0% (1,448/1,834) of patients.

### Safety

The AEs and ADRs are summarized in Table 2. The common AEs and ADRs according to IPSS risk categories are shown in Supplementary Table 1. The common AEs were pneumonia, transformation to AML, MDS progression, cardiac failure and sepsis. The common ADRs were transformation to AML, death, pneumonia and eosinophil count increased. These events were also observed across different IPSS risk categories.

**Table 2:** Safety summary of AEs and ADRs

AE		n	(%)
Number of patients		1834	-
AEs		1377	(75.1)
Common AEs (≥ 5.0%)	Pneumonia	304	(16.6)
	Transformation to acute myeloid leukaemia	165	(9.0)
	Myelodysplastic syndromes progression	137	(7.5)
	Cardiac failure	102	(5.6)
	Sepsis	97	(5.3)
SAEs		1172	(63.9)
Common SAEs (≥ 5.0%)	Pneumonia	272	(14.8)
	Transformation to acute myeloid leukaemia	161	(8.8)
	Myelodysplastic syndromes progression	120	(6.5)
	Sepsis	95	(5.2)
	Cardiac failure	92	(5.0)
AEs with fatal outcome		904	(49.3)
Common AEs (≥ 5.0%)	Transformation to acute myeloid leukaemia	133	(7.3)
	Pneumonia	122	(6.7)
	Myelodysplastic syndromes progression	100	(5.5)
ADR		n	(%)
Number of patients		1834	-
ADRs		414	(22.6)
Common ADRs (≥ 1.0%)	Transformation to acute myeloid leukaemia	40	(2.2)
	Death	37	(2.0)
	Pneumonia	24	(1.3)
	Eosinophil count increased	23	(1.3)
SADRs		234	(12.8)
Common SADRs (≥ 1.0%)	Transformation to acute myeloid leukaemia	39	(2.1)
	Death	37	(2.0)
	Pneumonia	21	(1.1)
ADRs with fatal outcome		141	(7.7)
Common ADRs (≥ 1.0%)	Death	37	(2.0)
	Transformation to acute myeloid leukaemia	30	(1.6)

MedDRA ver. 27.0

*ADR* adverse drug reaction, *AE* adverse event, *SADR* serious adverse drug reaction, *SAE* serious adverse event

ADRs associated with the onset or progression of malignant tumors (corresponding to Standardised MedDRA Queries: “Malignant tumours,” Preferred Term (PT): “Malignant neoplasm progression,” “Neoplasm progression,” and “Disease progression” in MedDRA/J version 27.0) were examined and categorized by the presence or absence of histories or comorbidities of solid tumors. Most of those ADRs (causal relationship with DA: not ruled out) were AML-related ADRs associated with the progression of MDS (corresponding to High Level Term: “Leukaemias acute myeloid,” PT: “Acute leukaemia,” “Myelodysplastic syndrome transformation,” “Leukaemia,” and “Myeloid leukaemia” in MedDRA/J version 27.0). ADRs associated with the onset or progression of malignant tumors other than AML (causal relationship with DA: not ruled out) were lung neoplasm malignant in one patient with a history or comorbidity of solid tumors, and blastic plasmacytoid dendritic cell neoplasia and lymphoma in one patient each with no history or comorbidity of solid tumors.

### Effectiveness

**Fig. 2:**
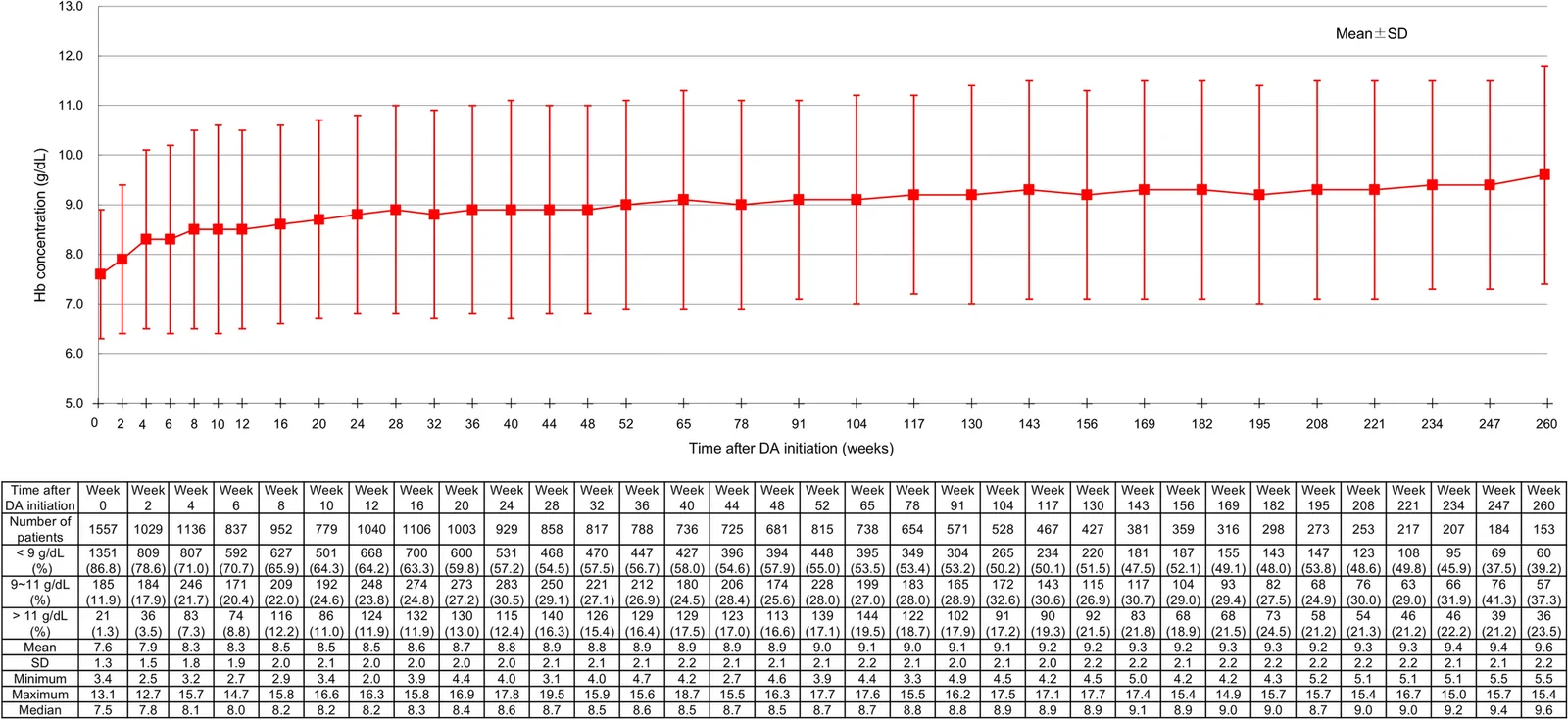
Time course of mean Hb concentration after DA initiation. Among patients in the effectiveness analysis population, the mean ± SD of Hb concentration is plotted at each time point up to 260 weeks after DA initiation for those in whom a transient increase in Hb due to red blood cell transfusion was considered unlikely (patients assessed more than 7 days after transfusion; however, Hb values on the day of re-transfusion were included if re-transfusion occurred within 7 days of the initial transfusion, as the transfusion effect at that point was deemed insufficient). *DA* darbepoetin alfa, *Hb* hemoglobin, *SD* standard deviation

The mean Hb concentrations were 7.6 g/dL at baseline, 8.3–8.9 g/dL at 4–48 weeks, and 9.0–9.6 g/dL at 52–260 weeks, showing a trend toward higher mean Hb concentrations after long-term treatment (Fig. 2).

The post-administration red blood cell transfusion status stratified by pre-administration transfusion history is shown in Table 3. Among red blood cell transfusion-dependent patients (n = 909), the highest annual proportion of transfusion independence was 40.9% at 4–5 years. Among red blood cell transfusion-independent patients (n = 911), the annual proportion of transfusion avoidance was approximately 60% (58.5%–64.9%) at all time points and remained at 63.7% at 4–5 years.

**Table 3:** Post-administration red blood cell transfusion status according to pre-administration transfusion history

Transfusion history within 56 days before DA initiation	Proportion of patients receiving red blood cell transfusion at each time point, n (%)												
		Up to 1 year		1–2 years		2–3 years		3–4 years		4–5 years		Final evaluation	
Yes (n = 909)	No	215	(23.7%)	108	(31.1%)	63	(33.2%)	49	(39.2%)	36	(40.9%)	225	(24.8%)
	Yes	693	(76.2%)	228	(65.7%)	116	(61.1%)	68	(54.4%)	49	(55.7%)	660	(72.6%)
	Unknown	1	(0.1%)	11	(3.2%)	11	(5.8%)	8	(6.4%)	3	(3.4%)	24	(2.6%)
No (n = 911)	No	591	(64.9%)	305	(61.0%)	193	(58.5%)	137	(59.1%)	100	(63.7%)	484	(53.1%)
	Yes	311	(34.1%)	171	(34.2%)	121	(36.7%)	86	(37.1%)	56	(35.7%)	384	(42.2%)
	Unknown	9	(1.0%)	24	(4.8%)	16	(4.8%)	9	(3.9%)	1	(0.6%)	43	(4.7%)

*DA* darbepoetin alfa

**Fig. 3:**
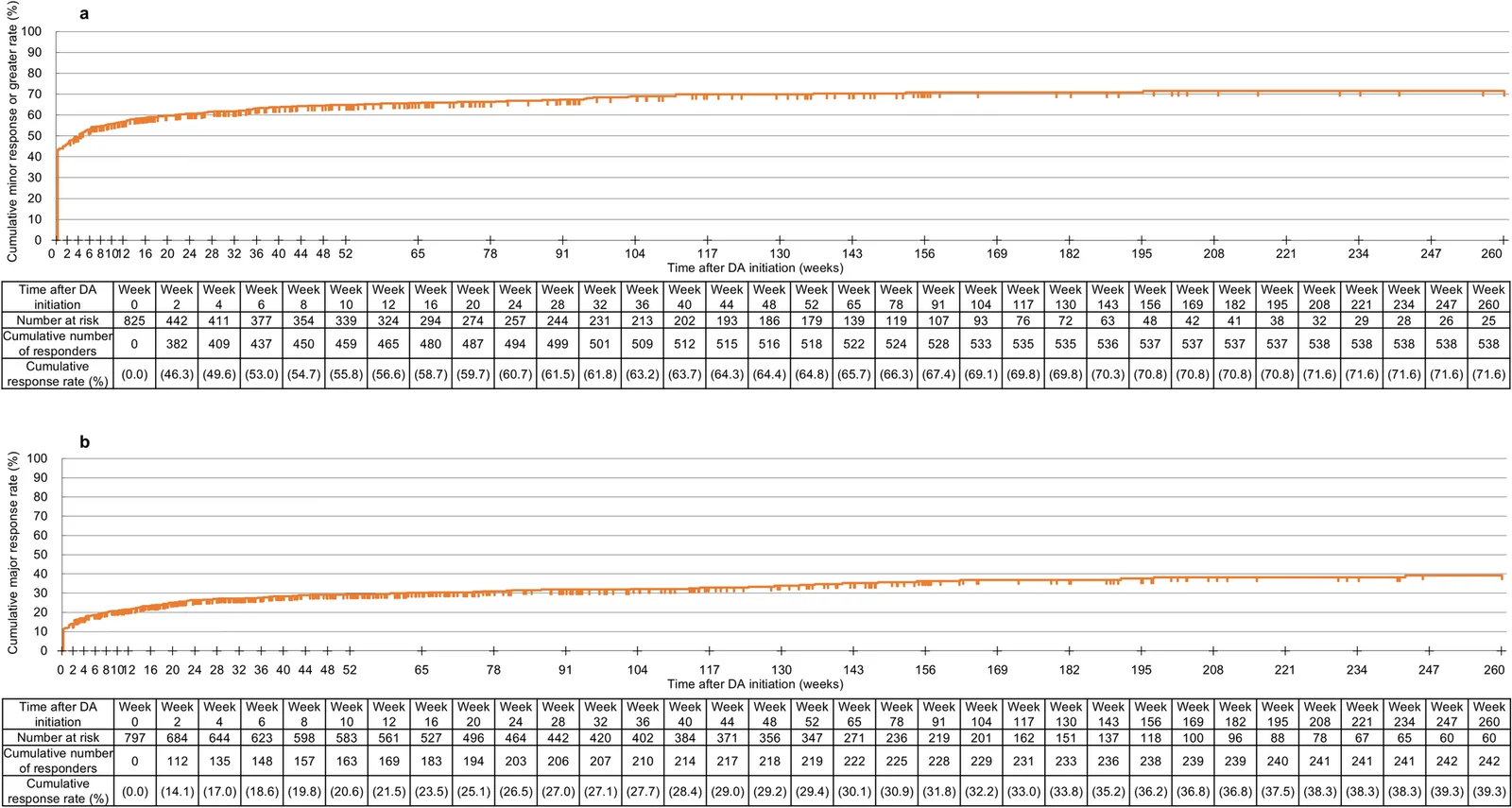
Cumulative erythroid response curves for a minor response or greater (**a**) and for a major response (**b**). Among patients in the effectiveness analysis population, cumulative erythroid response curves were generated for those with evaluable data for the erythroid response. The curves for a minor response or greater (**a**) and for a major response (**b**) at each time point up to 260 weeks after DA initiation are shown. *DA* darbepoetin alfa

Minor and major erythroid responses are summarized in Table 4. In the effectiveness analysis set (n = 1,821), 65.2% (538/825) of patients showed a minor response or greater, with the red blood cell transfusion volume decreased by ≥ 50% compared with baseline. In terms of major responses, 30.4% (242/797) of patients achieved transfusion independence with maximum Hb concentration increases of ≥ 1.0 g/dL compared with baseline. In terms of IPSS risk categories, the highest percentages of patients with a minor response or greater and a major response were all low-risk patients. When erythroid responses were evaluated at specific time points, the proportions achieving a minor response or greater at weeks 8 and 52 (n = 825) were 54.5% and 62.8%, and those achieving a major response (n = 797) were 19.7% and 27.5%, respectively. In addition, the cumulative erythroid response curves for a minor response or greater and for a major response (Fig. 3a and 3b) illustrate the proportion of patients who had achieved a response by each time point. The median time to response was 2 days for a minor response or greater and 16 days for a major response, and the cumulative proportions of patients achieving a response by weeks 8, 52, and 260 were 54.7%, 64.8%, and 71.6% for a minor response or greater (number at risk: 354/179/25), and 19.8%, 29.4%, and 39.3% for a major response (number at risk: 598/347/60).

**Table 4:** Erythroid response (minor, major)

Erythroid response	IPSS risk category	Effectiveness analysis population, n	Eligible patients, n	Observed response, n (%)		95% CI
Minor response^a^ or greater^b^	Overall	1821	825	538	(65.2%)	61.9%–68.5%
	Low	718	267	200	(74.9%)	69.3%–80.0%
	Intermediate-1	825	413	261	(63.2%)	58.3%–67.9%
	Intermediate-2	140	73	40	(54.8%)	42.7%–66.5%
	High	50	32	12	(37.5%)	21.1%–56.3%
	Unknown/not recorded	88	40	25	(62.5%)	45.8%–77.3%
Major response^c^	Overall	1821	797	242	(30.4%)	27.2%–33.7%
	Low	718	250	89	(35.6%)	29.7%–41.9%
	Intermediate-1	825	400	120	(30.0%)	25.5%–34.8%
	Intermediate-2	140	79	17	(21.5%)	13.1%–32.2%
	High	50	33	6	(18.2%)	7.0%–35.5%
	Unknown/not recorded	88	35	10	(28.6%)	14.6%–46.3%

*CI* confidence interval, *DA* darbepoetin alfa, *Hb*, hemoglobin, *IPSS* International Prognostic Scoring System

^a^Red blood cell transfusion volume for 56 consecutive days after DA initiation decreased by ≥ 50% compared with baseline. ^b^Patients with minor or major response. ^c^Patients not requiring red blood cell transfusion (withdrawal from red blood cell transfusion dependence) for at least 56 consecutive days after DA initiation and highest Hb concentration during withdrawal period increased by ≥ 1.0 g/dL compared with baseline

The erythroid responses by patient background are summarized. For prior ESA treatment status, the proportion of patients achieving minor response or greater/major response at weeks 8 and 52 was as follows: no prior ESA treatment (n = 763/745), overall response 65.4%/30.1% (week 8: 54.7%/19.6%, week 52: 62.8%/27.0%); prior ESA treatment (n = 61/51), overall response 63.9%/35.3% (week 8: 54.1%/21.6%, week 52: 63.9%/35.3%). Although the number of evaluable cases was limited, the proportion of patients achieving minor response or greater/major response at weeks 8 and 52 by pre-treatment EPO concentration were as follows: < 200 mIU/mL (n = 146/147), overall response 74.0%/36.1% (week 8: 65.1%/28.6%, week 52: 73.3%/34.7%); 200– < 500 mIU/mL (n = 56/55), overall response 58.9%/30.9% (week 8: 53.6%/16.4%, week 52: 58.9%/29.1%); ≥ 500 mIU/mL (n = 67/69), overall response 47.8%/15.9% (week 8: 34.3%/8.7%, week 52: 44.8%/14.5%). Additionally, although the number of evaluable cases was limited, the proportions of patients achieving minor response or greater/major response at weeks 8 and 52 by major karyotype were as follows: del(20q) (n = 56/52), overall response 67.9%/30.8% (week 8: 60.7%/15.4%, week 52: 67.9%/30.8%); -Y (n = 61/58), overall response 63.9%/37.9% (week 8: 54.1%/27.6%, week 52: 59.0%/34.5%); + 8 (n = 52/54), overall response 61.5%/18.5% (week 8: 55.8%/13.0%, week 52: 57.7%/18.5%); -5 or del(5q) (n = 59/62), overall response 49.2%/14.5% (week 8: 39.0%/12.9%, week 52: 45.8%/14.5%); -7 or del(7q) (n = 43/47), overall response 34.9%/10.6% (week 8: 30.2%/10.6%, week 52: 34.9%/10.6%); complex karyotypes (≥ 3 abnormalities) (n = 80/85), overall response 45.0%/14.1% (week 8: 40.0%/11.8%, week 52: 43.8%/14.1%).

The clinical global impression assessed by investigators is shown in Table 5. In all IPSS risk categories, a trend toward higher clinical global impression was observed as DA treatment progressed. Higher IPSS risk was associated with a lower proportion of effective cases in terms of the clinical global impression at the final evaluation and a higher proportion of ineffective cases.

**Table 5:** Clinical global impression assessed by investigators

IPSS risk category	Evaluation time point after DA initiation	Number of patients	Clinical global impression							
			Effective n (%)		Ineffective n (%)		Unable to determine n (%)		Unknown/not recorded n (%)	
Overall	Up to 1 year	1821	951	(52.2%)	634	(34.8%)	234	(12.9%)	2	(0.1%)
	1–2 years	843	596	(70.7%)	160	(19.0%)	86	(10.2%)	1	(0.1%)
	2–3 years	517	383	(74.1%)	77	(14.9%)	57	(11.0%)	0	(0.0%)
	3–4 years	355	277	(78.0%)	40	(11.3%)	35	(9.9%)	3	(0.8%)
	4–5 years	245	196	(80.0%)	23	(9.4%)	26	(10.6%)	0	(0.0%)
	Final evaluation	1821	760	(41.7%)	748	(41.1%)	307	(16.9%)	6	(0.3%)
Low	Up to 1 year	718	459	(63.9%)	187	(26.0%)	71	(9.9%)	1	(0.1%)
	1–2 years	400	295	(73.8%)	65	(16.3%)	40	(10.0%)	0	(0.0%)
	2–3 years	270	201	(74.4%)	37	(13.7%)	32	(11.9%)	0	(0.0%)
	3–4 years	193	146	(75.6%)	19	(9.8%)	27	(14.0%)	1	(0.5%)
	4–5 years	148	116	(78.4%)	14	(9.5%)	18	(12.2%)	0	(0.0%)
	Final evaluation	718	370	(51.5%)	234	(32.6%)	112	(15.6%)	2	(0.3%)
Intermediate-1	Up to 1 year	825	401	(48.6%)	318	(38.5%)	106	(12.8%)	0	(0.0%)
	1–2 years	371	262	(70.6%)	73	(19.7%)	35	(9.4%)	1	(0.3%)
	2–3 years	216	163	(75.5%)	33	(15.3%)	20	(9.3%)	0	(0.0%)
	3–4 years	145	120	(82.8%)	16	(11.0%)	7	(4.8%)	2	(1.4%)
	4–5 years	91	74	(81.3%)	9	(9.9%)	8	(8.8%)	0	(0.0%)
	Final evaluation	825	321	(38.9%)	368	(44.6%)	133	(16.1%)	3	(0.4%)
Intermediate-2	Up to 1 year	140	38	(27.1%)	78	(55.7%)	24	(17.1%)	0	(0.0%)
	1–2 years	24	11	(45.8%)	10	(41.7%)	3	(12.5%)	0	(0.0%)
	2–3 years	10	7	(70.0%)	2	(20.0%)	1	(10.0%)	0	(0.0%)
	3–4 years	5	2	(40.0%)	3	(60.0%)	0	(0.0%)	0	(0.0%)
	4–5 years	1	1	(100.0%)	0	(0.0%)	0	(0.0%)	0	(0.0%)
	Final evaluation	140	29	(20.7%)	86	(61.4%)	25	(17.9%)	0	(0.0%)
High	Up to 1 year	50	15	(30.0%)	23	(46.0%)	12	(24.0%)	0	(0.0%)
	1–2 years	11	5	(45.5%)	5	(45.5%)	1	(9.1%)	0	(0.0%)
	2–3 years	3	0	(0.0%)	2	(66.7%)	1	(33.3%)	0	(0.0%)
	3–4 years	0		-		-		-		-
	4–5 years	0		-		-		-		-
	Final evaluation	50	12	(24.0%)	26	(52.0%)	12	(24.0%)	0	(0.0%)
Unknown/not recorded	Up to 1 year	88	38	(43.2%)	28	(31.8%)	21	(23.9%)	1	(1.1%)
	1–2 years	37	23	(62.2%)	7	(18.9%)	7	(18.9%)	0	(0.0%)
	2–3 years	18	12	(66.7%)	3	(16.7%)	3	(16.7%)	0	(0.0%)
	3–4 years	12	9	(75.0%)	2	(16.7%)	1	(8.3%)	0	(0.0%)
	4–5 years	5	5	(100.0%)	0	(0.0%)	0	(0.0%)	0	(0.0%)
	Final evaluation	88	28	(31.8%)	34	(38.6%)	25	(28.4%)	1	(1.1%)

*DA* darbepoetin alfa, *IPSS* International Prognostic Scoring System

### OS and LFS

**Fig. 4:**
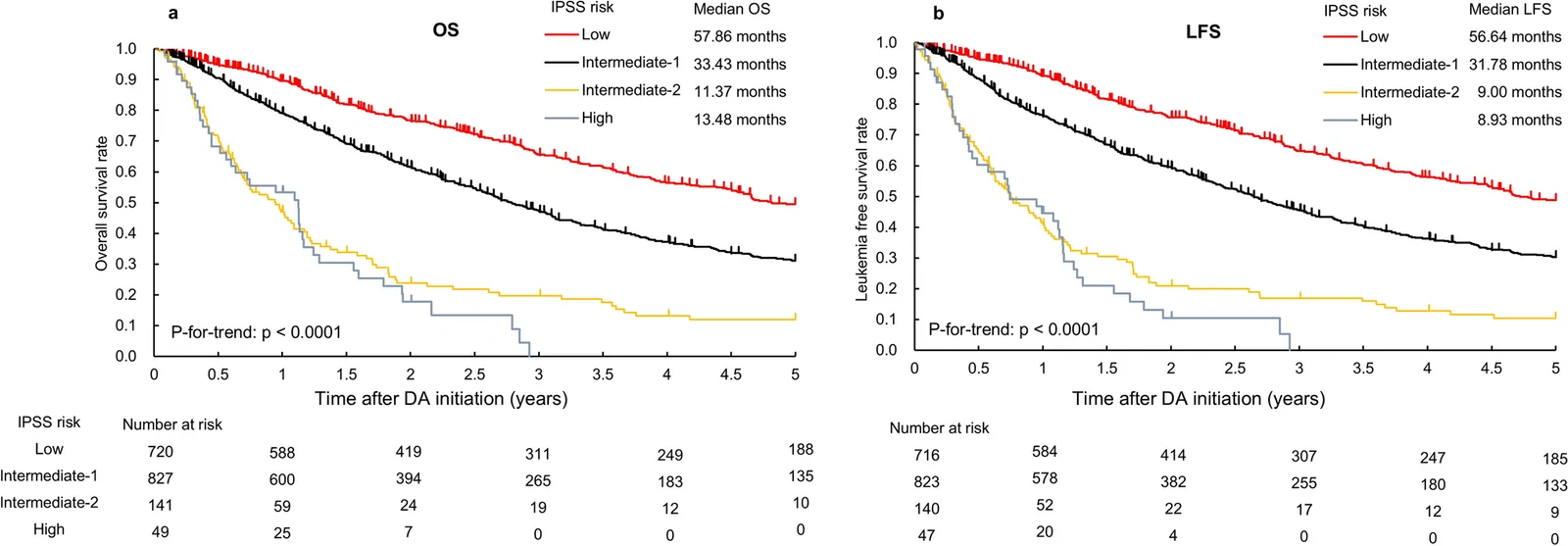
Kaplan–Meier survival curves for OS (**a**) and LFS (**b**) after DA initiation according to IPSS risk categories. Patients with available IPSS risk classification in the prognostic factor analysis population for mortality (**a**) and for death and transformation to AML (**b**) were analyzed. The IPSS risk categories (Low-, Intermediate-1-, Intermediate-2-, and High) were compared using log-rank tests. *DA* darbepoetin alfa, *IPSS* International Prognostic Scoring System, *LFS* leukemia-free survival, *OS* overall survival

**Fig. 5:**
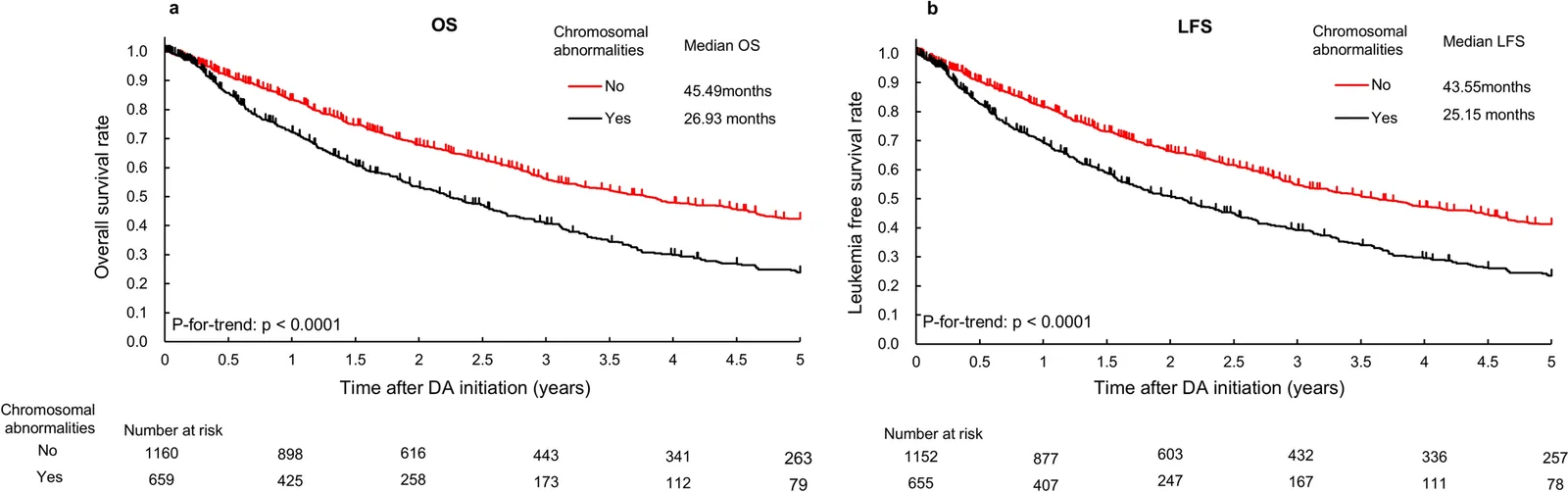
Kaplan–Meier survival curves for OS (**a**) and LFS (**b**) after DA initiation according to chromosomal abnormalities. Patients with available chromosomal abnormality data in the prognostic factor analysis populations for mortality (**a**) and for death and transformation to AML (**b**) were analyzed. Patients with and without chromosomal abnormalities were compared using log-rank tests.* DA* darbepoetin alfa, *LFS* leukemia-free survival, *OS* overall survival

**Fig. 6:**
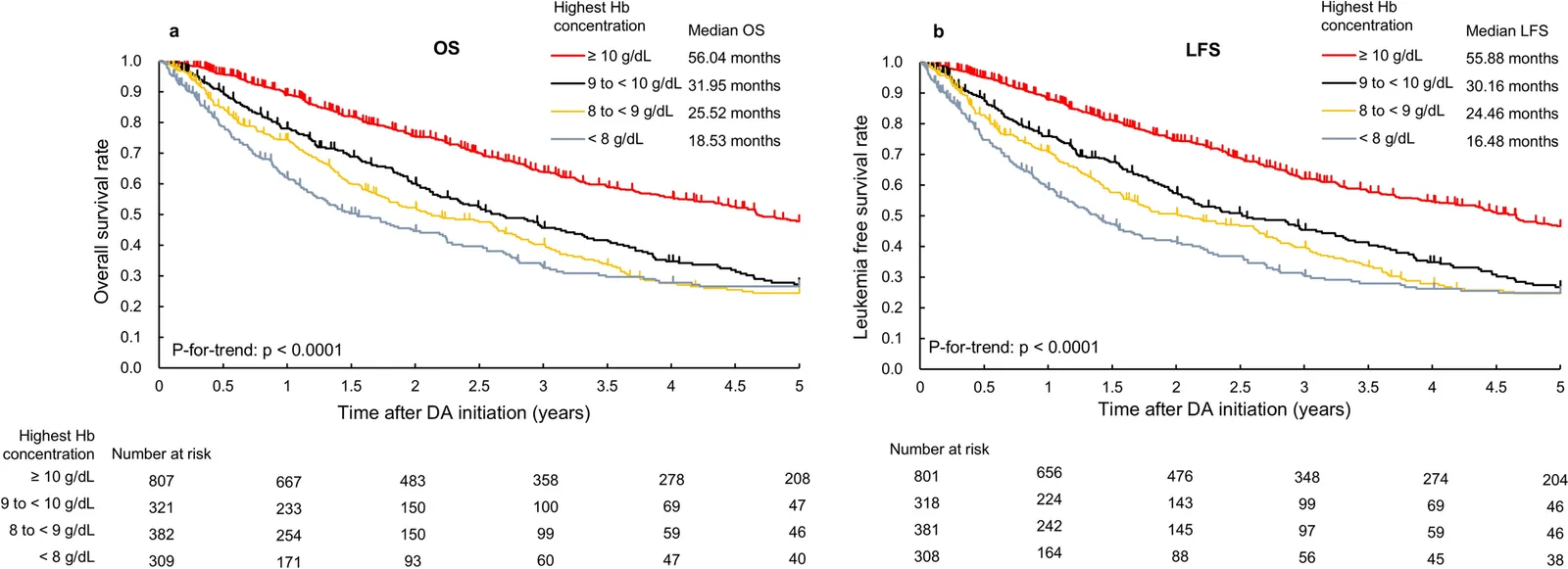
Kaplan–Meier survival curves for OS (**a**) and LFS (**b**) after DA initiation according to highest Hb concentration. Patients with available Hb concentration data in the prognostic factor analysis populations for mortality (**a**) and for death and transformation to AML (**b**) were analyzed. Groups with Hb ≥ 10 g/dL, 9 to < 10 g/dL, 8 to < 9 g/dL, and < 8 g/dL after DA treatment were compared using log-rank tests. *DA* darbepoetin alfa, *Hb* hemoglobin, *LFS* leukemia-free survival, *OS* overall survival

**Fig. 7:**
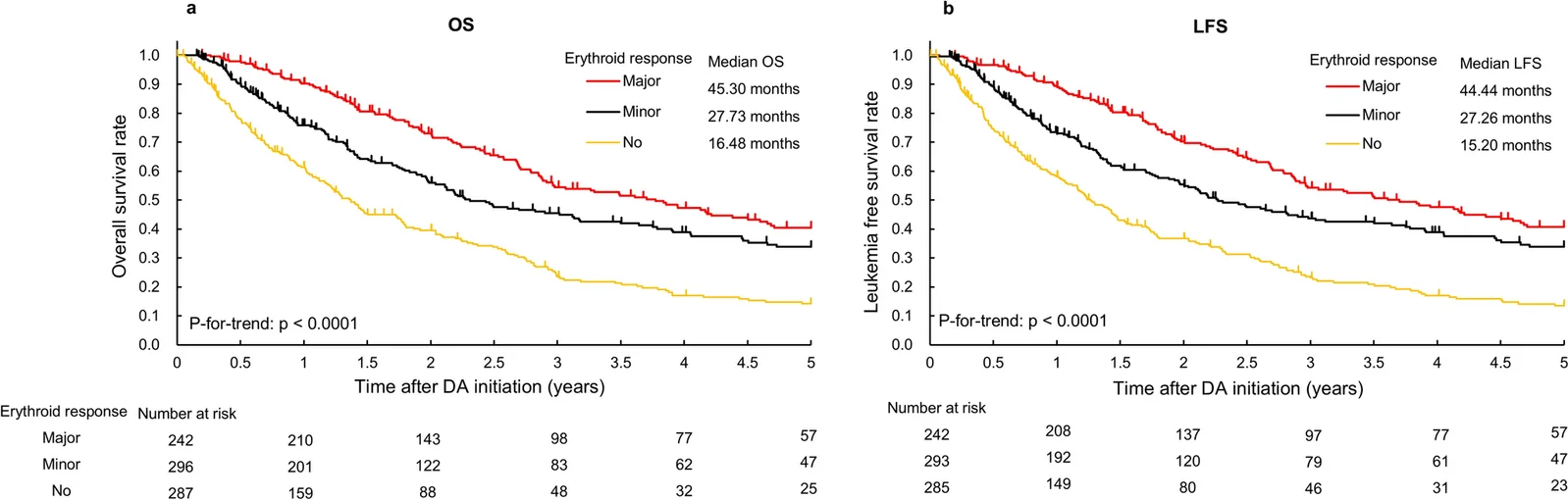
Kaplan–Meier survival curves for OS (**a**) and LFS (**b**) after DA initiation according to erythroid response. Patients with available erythroid response data in the prognostic factor analysis populations for mortality (**a**) and for death and transformation to AML (**b**) were analyzed. Groups with major, minor, and no erythroid response were compared using log-rank tests. *DA* darbepoetin alfa, *LFS* leukemia-free survival, *OS* overall survival

Higher IPSS risk was associated with significantly shorter median OS/LFS (*p* < 0.0001) and lower 5-year OS/LFS rates (Fig. 4a, b). The 5-year OS/LFS rates in 1,826/1,814 patients in the IPSS Low, Intermediate-1, Intermediate-2, and High risk categories were 49.5%/48.8%, 31.1%/30.2%, 11.9%/10.4%, and 0%/0%, respectively. Patients with chromosomal abnormalities had significantly shorter median OS/LFS (*p* < 0.0001) and lower 5-year OS/LFS rates (Fig. 5a, b). The 5-year OS/LFS rates in 1,826/1,814 patients with normal karyotype and any chromosomal abnormalities were 42.4%/41.3% and 24.0%/23.6%, respectively. In addition, the 5-year OS/LFS rates by major karyotype were as follows: del(20q) (n = 134/134), 40.8%/40.7%; -Y (n = 141/141), 30.3%/30.4%; + 8 (n = 109/109), 20.3%/21.3%; -5 or del(5q) (n = 90/88), 9.9%/9.8%; -7 or del(7q) (n = 76/73), 3.4%/2.6%; and complex karyotypes (≥ 3 abnormalities) (n = 142/140), 7.0%/7.3%. Lower levels of peak post-DA Hb concentration were associated with significantly shorter median OS/LFS (*p* < 0.0001) and lower 5-year OS/LFS rates (Fig. 6a, b). The 5-year OS/LFS rates in 1,826/,1814 patients at ≥ 10, 9 to < 10, 8 to < 9, and < 8 g/dL of the highest Hb concentration were 47.8%/46.6%, 27.2%/26.7%, 24.4%/24.7%, and 26.5%/24.9%, respectively. Patients without an erythroid response had significantly shorter median OS/LFS (*p* < 0.0001) and lower 5-year OS/LFS rates compared with those with a major or minor erythroid response (Fig. 7a, b). The 5-year OS/LFS rates in 1,826/1,814 patients with major erythroid, minor erythroid, and no erythroid response were 40.5%/40.8%, 33.9%/33.9%, and 14.2%/13.5%, respectively.

### Prognostic factor analysis

Among the prognostic factor analysis of mortality (n = 1,826) and death and transformation to AML (n = 1,814) populations, multivariable analyses identified IPSS risk category, age, cytogenetic findings, pre-treatment platelet count, and a history or comorbidity of malignancy as significant factors associated with a poor prognosis in both groups (*p* < 0.01). Higher IPSS risk, older age, the presence of chromosomal abnormalities, lower pre-treatment platelet count, and a history or comorbidity of malignant tumors were associated with increased hazard ratios in both analyses (Tables 6 and 7).

**Table 6:** Background factors associated with death in population for prognostic factor analysis

Item	Classification	Number of patients	Number of deaths n (%)		Cox proportional hazards model								
					Analysis set^a^	Number of deaths n (%)		Univariable analysis			Multivariable analysis		
								Hazard ratio	95% CI	p-value	Hazard ratio	95% CI	*p*-value
Analysis population	-	1826	907	(49.7%)	1451	730	(50.3%)	-	-	-	-	-	-
IPSS risk category immediately before DA initiation	Low	720	268	(37.2%)	577	216	(37.4%)	REF	-	-	REF	-	-
	Intermediate-1	827	441	(53.3%)	708	385	(54.4%)	1.82	1.54–2.15	< 0.0001	1.46	1.21–1.75	< 0.0001
	Intermediate-2	141	105	(74.5%)	121	92	(76.0%)	4.79	3.74–6.13	< 0.0001	3.27	2.48–4.32	< 0.0001
	High	50	40	(80.0%)	45	37	(82.2%)	6.00	4.21–8.56	< 0.0001	4.37	3.00–6.35	< 0.0001
	Unknown/not recorded	88	53	(60.2%)	-	-	-	-	-	-	-	-	-
Age (years)	< 70	316	119	(37.7%)	246	96	(39.0%)	0.71	0.56–0.89	0.0038	0.68	0.54–0.86	0.0011
	70 to 79	644	322	(50.0%)	539	272	(50.5%)	REF	-	-	REF	-	-
	≥ 80	866	466	(53.8%)	666	362	(54.4%)	1.34	1.14–1.57	0.0003	1.35	1.15–1.59	0.0002
Chromosomal abnormalities	No	1161	521	(44.9%)	884	395	(44.7%)	REF	-	-	REF	-	-
	Yes	659	383	(58.1%)	567	335	(59.1%)	1.73	1.49–2.00	< 0.0001	1.32	1.13–1.54	0.0004
	Unknown/not recorded	6	3	(50.0%)	-	-	-	-	-	-	-	-	-
Pre-treatment Hb concentration (g/dL)	< 9	1403	726	(51.7%)	1259	652	(51.8%)	REF	-	-	REF	-	-
	9 to < 11	198	86	(43.4%)	167	70	(41.9%)	0.72	0.56–0.92	0.0099	0.84	0.65–1.08	0.1648
	≥ 11	28	9	(32.1%)	25	8	(32.0%)	0.45	0.23–0.91	0.0260	0.63	0.31–1.27	0.1961
	Unknown/not recorded	197	86	(43.7%)	-	-	-	-	-	-	-	-	-
Pre-treatment neutrophil count (× 10^3^/µL)	< 1.8	756	433	(57.3%)	718	407	(56.7%)	REF	-	-	REF	-	-
	≥ 1.8	776	345	(44.5%)	733	323	(44.1%)	0.75	0.65–0.87	< 0.0001	0.96	0.82–1.13	0.6357
	Unknown/not recorded	294	129	(43.9%)	-	-	-	-	-	-	-	-	-
Pre-treatment platelet count (× 10^3^/µL)	< 100	671	407	(60.7%)	583	355	(60.9%)	1.83	1.58–2.12	< 0.0001	1.49	1.27–1.75	< 0.0001
	≥ 100	942	410	(43.5%)	868	375	(43.2%)	REF	-	-	REF	-	-
	Unknown/not recorded	213	90	(42.3%)	-	-	-	-	-	-	-	-	-
History or comorbidity of malignant tumors	No	1497	719	(48.0%)	1179	569	(48.3%)	REF	-	-	REF	-	-
	Yes	329	188	(57.1%)	272	161	(59.2%)	1.40	1.18–1.67	0.0002	1.31	1.10–1.57	0.0027

*CI* confidence interval, *DA* darbepoetin alfa, *Hb* hemoglobin, *n.s.* not significant, *REF* reference

^***^p < 0.001, **p < 0.01, *p < 0.05, n.s. *p* ≥ 0.05

^a^Patients with no missing values in the explanatory variables

**Table 7:** Background factors associated with death and transformation to AML in population for prognostic factor analysis

Item	Classification	Number of patients	Number of patients with death or AML n (%)		Cox proportional hazards model											
					Analysis set^a^		Number of patients with death or AML n (%)			Univariable analysis				Multivariable analysis		
										Hazard ratio		95% CI	p-value	Hazard ratio	95% CI	*p*-value
Analysis population	-	1814	926	(51.0%)	1445		747	(51.7%)		-		-	-	-	-	-
IPSS risk category immediately before DA initiation	Low	716	271	(37.8%)	575		218	(37.9%)		REF		-	-	REF	-	-
	Intermediate-1	823	452	(54.9%)	706		395	(55.9%)		1.88		1.59–2.22	< 0.0001	1.50	1.25–1.80	< 0.0001
	Intermediate-2	140	110	(78.6%)	120		97	(80.8%)		5.24		4.11–6.67	< 0.0001	3.56	2.71–4.69	< 0.0001
	High	48	40	(83.3%)	44		37	(84.1%)		6.64		4.66–9.47	< 0.0001	5.00	3.45–7.26	< 0.0001
	Unknown/not recorded	87	53	(60.9%)	-		-	-		-		-	-	-	-	-
Age (years)	< 70	312	128	(41.0%)	244		103	(42.2%)		0.75		0.60–0.94	0.0120	0.71	0.56–0.89	0.0032
	70 to 79	642	329	(51.2%)	539		279	(51.8%)		REF		-	-	REF	-	-
	≥ 80	860	469	(54.5%)	662		365	(55.1%)		1.29		1.10–1.51	0.0014	1.32	1.12–1.54	0.0007
Chromosomal abnormalities	No	1153	533	(46.2%)	880		405	(46.0%)		REF		-	-	REF	-	-
	Yes	655	390	(59.5%)	565		342	(60.5%)		1.73		1.50–2.00	< 0.0001	1.31	1.13–1.53	0.0005
	Unknown/not recorded	6	3	(50.0%)	-		-	-		-		-	-	-	-	-
Pre-treatment Hb concentration (g/dL)	< 9	1392	741	(53.2%)	1253		666	(53.2%)		REF		-	-	REF	-	-
	9 to < 11	198	89	(44.9%)	167		73	(43.7%)		0.74		0.58–0.94	0.0129	0.87	0.68–1.11	0.2479
	≥ 11	28	9	(32.1%)	25		8	(32.0%)		0.44		0.22–0.89	0.0213	0.63	0.31–1.28	0.2019
	Unknown/not recorded	196	87	(44.4%)	-		-	-		-		-	-	-	-	-
Pre-treatment neutrophil count (× 10^3^/µL)	< 1.8	751	445	(59.3%)	714		419	(58.7%)		REF		-	-	REF	-	-
	≥ 1.8	774	350	(45.2%)	731		328	(44.9%)		0.72		0.62–0.83	< 0.0001	0.93	0.79–1.08	0.3400
	Unknown/not recorded	289	131	(45.3%)	-		-	-		-		-	-	-	-	-
Pre-treatment platelet count (× 10^3^/µL)	< 100	664	415	(62.5%)	578		363	(62.8%)		1.85		1.60–2.14	< 0.0001	1.47	1.26–1.72	< 0.0001
	≥ 100	940	419	(44.6%)	867		384	(44.3%)		REF		-	-	REF	-	-
	Unknown/not recorded	210	92	(43.8%)	-		-	-		-		-	-	-	-	-
History or comorbidity of malignant tumors	No	1487	730	(49.1%)	1174		581	(49.5%)		REF		-	-	REF	-	-
	Yes	327	196	(59.9%)	271		166	(61.3%)		1.41		1.19–1.68	< 0.0001	1.30	1.09–1.55	0.0034

*AML* acute myeloid leukemia, *CI* confidence interval, *DA* darbepoetin alfa, *Hb* hemoglobin, *n.s.* not significant, *REF* reference

^***^p < 0.001, **p < 0.01, *p < 0.05, n.s. *p* ≥ 0.05

^a^Patients with no missing values in the explanatory variables

## Discussion

This PMS evaluated the long-term safety and effectiveness of DA in patients with anemia associated with MDS under clinical conditions in Japan. No new concerns regarding safety or effectiveness were identified compared with previous clinical trials [7, 8].

The age distribution in this PMS (registration period: February 2015–June 2019; median age: 79.0 years) was similar to that in the Japan Adult Leukemia Study Group (JALSG) large-scale observational study (registration period: August 2011–January 2016; median age: 74 years) [9], suggesting no marked difference in age distribution compared with general MDS. The Japanese package insert for DA (product name: NESP) states that “the efficacy and safety of NESP in patients classified as intermediate‑2 or high risk by IPSS have not been established” [10], and the guidelines recommend DA administration for lower‑risk MDS (IPSS Low/Intermediate‑1) [1–4]. However, this PMS also enrolled patients with higher‑risk MDS (IPSS Intermediate‑2: 7.7% and High: 2.8%) in the real‑world clinical setting. The specific reasons for using DA in these patients are unclear, as its use was based on investigator judgment and information on the reasons for DA administration was not collected. Nevertheless, the primary target population of this PMS is lower‑risk MDS, for which DA use is guideline‑supported. Therefore, the results presented here for higher‑risk MDS simply reflect real‑world data collection and are not intended for drawing conclusions regarding the efficacy or safety of DA in higher‑risk disease.

The median administration period was approximately 1 year (330.0 days). Although a shorter treatment duration tended to be associated with increasing IPSS risk, some high-risk patients continued long-term administration, and 9.1% (166/1,833) of patients overall were still receiving DA at 5 years (260 weeks) after initiation. Given that the administration interval tended to be longer in the late stage than in the initial stage (mean administration interval: 1.4 weeks at 2 weeks, 2.0–2.7 weeks at 12–48 weeks, 3.0–4.0 weeks at 52–260 weeks after DA initiation), patients with long-term administration were considered to have effective treatment without requiring frequent dosing.

The incidence of ADRs did not exceed that reported at approval (22.6% vs. 34.6% [8]), and there were no marked differences in common AE/ADR profiles, regardless of IPSS risk category. The common ADRs with fatal outcomes were considered attributable to factors other than DA, such as underlying disease, advanced age, susceptibility to infection, medical history, and comorbidities, and none was specific to DA.

There were no significant differences in the incidence of ADRs associated with the onset or progression of malignant tumors, regardless of the presence or absence of a history or comorbidity of solid tumors; however, this study lacked a control group without DA, thus limiting the interpretation of the data. It is therefore unclear whether DA administration increases the risk of cancer progression or recurrence.

The mean Hb concentration tended to be higher in the late stage compared with the initial stage after DA initiation, maintaining a level that did not require dose escalation (mean Hb concentration ≥ 9.0 g/dL) after week 52 (mean Hb concentration: 7.6 g/dL at baseline, 8.3–8.9 g/dL at weeks 4–48, 9.0–9.6 g/dL at weeks 52–260). Ring sideroblasts (RS) represent a dysplastic feature highly specific to MDS. Luspatercept, approved in the United States and Europe in 2020 and in Japan in 2021, is an established treatment option for anemia in lower-risk MDS-RS [1–5]. We also evaluated the effect of RS status on the Hb response following DA administration. The mean Hb concentration at baseline in RS-positive patients (n = 118, corresponding to “refractory anemia with ringed sideroblasts [RARS]” and “refractory cytopenia with multilineage dysplasia [RCMD]-RS” in WHO classification version 3.0) was the same as that in the overall population (7.6 g/dL). The mean Hb concentration increased in RS-positive patients after DA initiation, but the increase was small (mean Hb concentration 8.0–9.2 g/dL at weeks 2–260 and ≥ 9.0 g/dL at only 2 time points: 9.1 g/dL at week 10 and 9.2 g/dL at week 44). Furthermore, comparing RS-positive and RS-negative patients (corresponding to “refractory anemia [RA],” “RCMD,” “refractory anemia with excess blasts [RAEB]-1,” “RAEB-2,” “myelodysplastic syndromes unclassifiable [MDS-U],” and “5q-syndrome” in the WHO classification version 3.0), there was a significant difference in the percentage of patients showing a major response between the two groups (RS-positive: 42.1%, RS-negative: 29.0%, *p* = 0.0490) (Supplementary Table 2). RS-positive patients, however, may be underestimated in Japan compared with Western countries, possibly because of unstable iron staining at diagnosis [11], and caution is therefore required when interpreting these results.

Direct comparisons between this PMS and the clinical trial for approval [7] were limited because of differences in patient numbers (1,821 vs 15), patient characteristics (patients in poor condition included vs. patients only with IPSS Low risk, Intermediate-1 risk, and PS 2 or less included), observation period (5 years vs. 16 weeks), and other conditions. Nevertheless, there were no significant differences in erythroid response (minor response or greater and major response) between this PMS and the clinical trial for approval (minor response or greater: 65.2% vs. 66.7%, major response: 30.4% vs. 33.3%) [7].

In this PMS, the erythroid response rates showed that a substantial proportion of patients had already responded at an early time point, with 54.5% achieving a minor response or greater and 19.7% achieving a major response by week 8 (overall rates: 65.2% and 30.4%), and the median times to achieving a minor response or greater and a major response were 2 days and 16 days, respectively. In our analysis of the results based on patient background information, the rates of minor response or greater and major response were broadly comparable between patients with and without prior ESA treatment (with vs. without ESA: 63.9%/35.3% vs. 65.4%/30.1%). Furthermore, lower EPO concentrations were associated with higher response rates (minor response or greater/major response for < 200 mIU/mL, 200– < 500 mIU/mL, and ≥ 500 mIU/mL: 74.0%/36.1%, 58.9%/30.9%, and 47.8%/15.9%), which is consistent with commonly reported findings [1–4].

Among patients who were red blood cell transfusion-dependent before DA initiation, the transfusion avoidance rate tended to increase as the administration period progressed (23.7%–40.9%). Additionally, a stable transfusion avoidance rate (approximately 60%) was maintained throughout the administration period, even in patients who were red blood cell transfusion-independent before DA initiation. These results demonstrate that long-term DA therapy maintained Hb levels and reduced the transfusion burden.

Although direct comparison is limited by differences in study design and patient characteristics, the 5-year OS (%) according to IPSS category was comparable to that in the JALSG large-scale observational study [9] (this PMS: 49.5% and 31.1% in patients with IPSS Low and Intermediate-1 risk; JALSG large-scale observational study: 32.3% in all patients and 45.3% in ESA-treated patients with IPSS-R lower risk group). Another observational cohort study of ESA exposure in lower-risk MDS patients reported no significant difference in AML transition rates between the ESA-exposed and -unexposed groups, and the median OS was significantly prolonged in the ESA-exposed group by 10.1 months compared with the unexposed group [12, 13]. The incidence rate of AML-related AEs in this PMS was comparable to the AML transition rate in the ESA-exposed group in the above cohort study (this PMS: 12.4%; cohort study: 17.5%), and the median OS was also comparable to that of the ESA-exposed group in the same cohort study (this PMS: IPSS Low: 57.86 months and Intermediate risk-1: 33.43 months; cohort study: 44.9 months). Notably, several studies have reported that ESA administration does not affect progression to AML [14–19]. In this PMS, the survival curves for the IPSS Intermediate-2 and High-risk categories closely overlapped. However, as mentioned earlier, Higher-risk MDS is not the main target of this PMS. Therefore, this overlap may reflect the relatively small number of patients in these subgroups and potential selection bias associated with the fact that DA-eligible patients may constitute a population in a clinically better condition.

Poor prognostic background factors detected in this PMS (higher IPSS risk, older age, chromosomal abnormalities, lower pre-treatment platelet count, and history or comorbidity of malignant tumors) were similar to those generally reported for MDS [1, 2], suggesting that worsening of the primary disease (MDS) because of these poor prognostic factors may have led to “death” or “death and transformation to AML,” regardless of DA treatment.

The prognosis according to karyotype in this PMS was generally consistent with commonly reported prognoses (good prognosis: del(20q), -Y, del(5q); poor prognosis: complex karyotype [≥ 3 abnormalities], chromosome 7 abnormalities) [1]; however, the prognosis for -5 or del(5q) was particularly unfavorable in this PMS (5-year OS/LFS rates: 9.9%/9.8%). This may be because approximately 53% of patients with -5 or del(5q) exhibited complex karyotypes (≥ 3 abnormalities) including -5 or del(5q), and the prognosis of these specific patients may have contributed to the overall poor prognosis in patients with -5 or del(5q). Although lenalidomide may have been withheld in cases with complex karyotypes (≥ 3 abnormalities), lenalidomide was only used in 18%, 16%, and 3% of patients with -5 or del(5q) before and after DA initiation and at both, respectively. Additional information on patients with -5 or del(5q) showed that a decline in efficacy over time was not observed, with minor response or greater and major response rates at week 52 slightly exceeding those at week 8 (39.0%/12.9% at week 8 vs. 45.8%/14.5% at week 52; n = 59/62). Among patients without complex karyotypes (≥ 3 abnormalities), response rates showed a similar pattern, with slightly higher rates at week 52 than at week 8 (48.1%/9.7% vs. 55.6%/12.9%; n = 27/31).

The highest Hb concentrations after DA initiation were associated with better OS/LFS, with approximately 47% of patients with a mean Hb concentration ≥ 10 g/dL surviving/being leukemia-free at 5 years. Patients with a minor or major erythroid response also showed more favorable OS/LFS compared with those without an erythroid response. Although these findings demonstrate a clear association between Hb improvement and survival outcomes, this relationship may partly reflect underlying disease biology, such as better bone marrow reserve or less aggressive disease in patients who achieve an erythroid response.

This PMS had several limitations. First, the quality of the PMS data was not as high as in clinical trials. Clinical trials involve direct inspection and verification of source documents to ensure their consistency, and queries are reliably addressed to collect and aggregate data with overall consistency. In contrast, PMS studies do not verify consistency between questionnaires and source documents. Furthermore, in some cases, data, including fixed entries obtained without follow-up responses, were collected and aggregated. Second, PMS studies have the potential to provide additional information on clinical utility, such as clinical outcomes and quality of life, that is not obtained in clinical trials [20]. Although this PMS also acquired valuable data on the clinical global impression assessment, the validity of the results relied solely on investigator assessments and was not evaluated strictly, uniformly, or objectively, as in prospective clinical trials. Finally, the RS-positive/negative classification in this PMS may not be entirely accurate. Although differences in effectiveness between RS-positive and RS-negative patients were considered an important point, RS-positive cases may have been underestimated in Japan because of inappropriate iron staining at diagnosis [11]. Caution is therefore required in interpreting these results. Because of this limitation, this paper did not include findings regarding RS-positivity/negativity in the Results section, and we have therefore only mentioned this aspect in the Discussion section after noting this point.

In conclusion, the safety profile and effectiveness of DA demonstrated in the present PMS were comparable to those in previous clinical trials [7, 8], indicating no new concerns regarding its safety and effectiveness. The poor prognostic factors detected in this PMS were similar to those generally reported for MDS [1, 2], suggesting that worsening of the primary disease (MDS) because of these poor prognostic factors may have led to “death” or “death and transformation to AML,” regardless of DA treatment. Among patients receiving DA treatment in this PMS, a higher Hb concentration and the presence of an erythroid response (minor, major) were associated with better OS/LFS. These associations suggest that achieving the treatment goals of improving anemia and reducing red blood cell transfusions requirements remains clinically meaningful; however, it is also likely that patients who respond to DA have more favorable baseline characteristics that contribute to both the hematologic response and improved survival. The findings from this large-scale real-world PMS may provide valuable guidance for clinicians considering DA therapy in patients with MDS-related anemia.

## Supplementary Information

Below is the link to the electronic supplementary material.

Supplementary file1 (DOCX 65 kb)

Supplementary file2 (PPTX 50 kb)

## Data Availability

Anonymized data obtained in the current survey and used for the analysis may be available upon reasonable request from a principal investigator to the corresponding author.
