# Supplementary material for: Darbepoetin alfa for the treatment of anemia in myelodysplastic syndromes: a post-marketing surveillance study in Japan
Source: Int J Hematol. 2026 May 18;124(4):502–20. doi: 10.1007/s12185-026-04227-w (PMC13638701; doi:10.1007/s12185-026-04227-w)
Supplement: Supplementary file 1 [file 12185_2026_4227_MOESM1_ESM.docx]

**Supplementary Tables**

**Supplementary Table 1** Summary of AEs and ADRs according to IPSS risk category

| AE | | | |
| --- | --- | --- | --- |
| IPSS risk category | Common PTs (≥ 5.0%) | n | (%) |
| Low  (n = 720) | Pneumonia | 82 | (11.4) |
|  | Myelodysplastic syndrome progression | 40 | (5.6) |
|  | Fall | 39 | (5.4) |
|  | Cardiac failure | 37 | (5.1) |
|  | Sepsis | 37 | (5.1) |
| Intermediate-1  (n = 827) | Pneumonia | 150 | (18.1) |
|  | Transformation to acute myeloid leukaemia | 86 | (10.4) |
|  | Myelodysplastic syndromes progression | 72 | (8.7) |
|  | Cardiac failure | 48 | (5.8) |
|  | Pyrexia | 47 | (5.7) |
| Intermediate-2  (n = 141) | Pneumonia | 41 | (29.1) |
|  | Transformation to acute myeloid leukaemia | 32 | (22.7) |
|  | Myelodysplastic syndromes progression | 17 | (12.1) |
|  | Febrile neutropenia | 16 | (11.3) |
|  | Pyrexia | 16 | (11.3) |
|  | Acute myeloid leukaemia | 14 | (9.9) |
|  | Sepsis | 12 | (8.5) |
|  | Cardiac failure | 11 | (7.8) |
|  | Death | 9 | (6.4) |
| High  (n = 52) | Pneumonia | 15 | (28.8) |
|  | Transformation to acute myeloid leukaemia | 15 | (28.8) |
|  | Acute myeloid leukaemia | 5 | (9.6) |
|  | Febrile neutropenia | 5 | (9.6) |
|  | Sepsis | 5 | (9.6) |
|  | Cardiac failure | 3 | (5.8) |
|  | Myelodysplastic syndromes progression | 3 | (5.8) |
| Unknown/not recorded  (n = 94) | Pneumonia | 16 | (17.0) |
|  | Transformation to acute myeloid leukaemia | 9 | (9.6) |
|  | Death | 8 | (8.5) |
|  | Fall | 6 | (6.4) |
|  | Acute myeloid leukaemia | 5 | (5.3) |
|  | Myelodysplastic syndromes progression | 5 | (5.3) |
| Overall  (n = 1834) | Pneumonia | 304 | (16.6) |
|  | Transformation to acute myeloid leukaemia | 165 | (9.0) |
|  | Myelodysplastic syndromes progression | 137 | (7.5) |
|  | Cardiac failure | 102 | (5.6) |
|  | Sepsis | 97 | (5.3) |
| ADR | | | |
| IPSS risk category | Common PTs (≥ 1.0%) | n | (%) |
| Low  (n = 720) | Death | 18 | (2.5) |
|  | Eosinophil count increased | 11 | (1.5) |
|  | Hypertension | 11 | (1.5) |
|  | Hyperuricaemia | 8 | (1.1) |
| Intermediate-1  (n = 827) | Transformation to acute myeloid leukaemia | 21 | (2.5) |
|  | Pneumonia | 17 | (2.1) |
|  | Death | 13 | (1.6) |
|  | Myelodysplastic syndromes progression | 11 | (1.3) |
|  | Eosinophil count increased | 10 | (1.2) |
|  | Pyrexia | 9 | (1.1) |
| Intermediate-2  (n = 141) | Transformation to acute myeloid leukaemia | 6 | (4.3) |
|  | Death | 4 | (2.8) |
|  | Pneumonia | 3 | (2.1) |
|  | Acute myeloid leukaemia | 2 | (1.4) |
|  | Cardiac failure | 2 | (1.4) |
|  | Febrile neutropenia | 2 | (1.4) |
|  | Urinary tract infection | 2 | (1.4) |
| High  (n = 52) | Common PTs (≥ 2.0%) | n | (%) |
|  | Acute myeloid leukaemia | 2 | (3.8) |
|  | Transformation to acute myeloid leukaemia | 2 | (3.8) |
| Unknown/not recorded  (n = 94) | Transformation to acute myeloid leukaemia | 6 | (6.4) |
|  | Death | 2 | (2.1) |
| Overall  (n = 1834) | Common PTs (≥ 1.0%) | n | (%) |
|  | Transformation to acute myeloid leukaemia | 40 | (2.2) |
|  | Death | 37 | (2.0) |
|  | Pneumonia | 24 | (1.3) |
|  | Eosinophil count increased | 23 | (1.3) |

MedDRA ver. 27.0

*ADR* adverse drug reaction, *AE* adverse event, *IPSS* International Prognostic Scoring System, *PT* Preferred Term

**Supplementary Table 2** Erythroid responses in RS-positive and RS-negative patients

| Erythroid response | RS | Responders, n | Non-responders, n | Eligible patients, n | Effectiveness | 95% CI^a^ | p value^b^ |
| --- | --- | --- | --- | --- | --- | --- | --- |
| Minor response^c^ or greater^d^ | Positive | 41 | 15 | 56 | 73.2% | 59.7%–84.2% | 0.1916 |
|  | Negative | 418 | 233 | 651 | 64.2% | 60.4%–67.9% |  |
| Major response^e^ | Positive | 24 | 33 | 57 | 42.1% | 29.1%–55.9% | 0.0490 |
|  | Negative | 183 | 449 | 632 | 29.0% | 25.4%–32.7% |  |

^a^ Calculated by Clopper–Pearson method; ^b^ Calculated by Fisher’s exact test

^c^ Red blood cell transfusion volume for 56 consecutive days after DA initiation decreased by ≥ 50% compared with baseline

^d^ Patients with minor or major response

^e^ Patients not requiring red blood cell transfusion (withdrawal from red blood cell transfusion dependence) for ≥ 56 consecutive days after DA initiation and highest Hb concentration during withdrawal period increased by ≥ 1.0 g/dL compared with baseline

*CI* confidence interval, *DA* darbepoetin alfa, *Hb* hemoglobin, *RS* ringed sideroblasts
